# Treatment of hyponatremia: comprehension and best clinical practice

**DOI:** 10.1007/s10157-024-02606-3

**Published:** 2025-01-23

**Authors:** Hirofumi Sumi, Naoto Tominaga, Yoshiro Fujita, Joseph G. Verbalis, the Electrolyte Winter Seminar Collaborative Group, Takuya Fujimaru, Yoshiro Fujita, Kazuhito Hirose, Kyogo Kawada, Toshiaki Monkawa, Masahiko Nagahama, Masatomo Ogata, Akihiro Ryuge, Yugo Shibagaki, Hideaki Shimizu, Hirofumi Sumi, Maho Terashita, Naoto Tominaga, Masahiko Yazawa

**Affiliations:** 1 Division of Nephrology and Hypertension, Kawasaki Municipal Tama Hospital, 1-30-37, Shukugawara, Tama-Ku, Kawasaki, Kanagawa 214-8525 Japan; 2 Division of Nephrology and Hypertension, Department of Internal Medicine, St. Marianna University School of Medicine, 2-16-1, Sugao, Miyamae-Ku, Kawasaki, Kanagawa 216-8511 Japan; 3 Department of Nephrology, Chubu Rosai Hospital, 1-10-6, Komei-Cho, Minato-Ku, Nagoya, Aichi 455-8530 Japan; 4 Division of Endocrinology and Metabolism, Department of Medicine, Georgetown University, 4000 Reservoir Rd NW, Washington, DC 20007 USA

**Keywords:** Hyponatremia, Treatment, Overly rapid correction, Osmotic demyelination syndrome

## Abstract

This review article series on water and electrolyte disorders is based on the ‘Electrolyte Winter Seminar’ held annually for young nephrologists in Japan. The seminar features dynamic case-based discussions, some of which are included as self-assessment questions in this series. The second article in this series focuses on treatment of hyponatremia, a common water and electrolyte disorder frequently encountered in clinical practice. Hyponatremia presents diagnostic challenges due to its various etiologies and the presence of co-morbidities that affect water and electrolyte homeostasis. Furthermore, limited evidence, including a lack of robust randomized controlled trials, complicates treatment decisions and increases the risk of poor outcomes from inappropriate management of both acute and chronic hyponatremia. This review provides a comprehensive overview of treatment of hyponatremia for better comprehension and improved clinical practice.

## Introduction

Hyponatremia is the most frequently encountered electrolyte disorder in daily clinical practice [1, 2]. The pathophysiology of hyponatremia is often difficult to interpret and evaluate due to multiple additional factors involved, which makes the choice of appropriate treatment challenging. Furthermore, there is an insufficiency of evidence-based treatment options for hyponatremia, which often necessitates a “trial-and-error approach” to treatment and may result in unfavorable outcomes in hyponatremic patients. With this background, new findings on treatment methods for the syndrome of inappropriate antidiuresis (SIAD), including vasopressin receptor antagonists (vaptans), hypertonic saline, and urea have recently been reported.

Clinicians should appropriately treat hyponatremia through appropriate evaluation based on pathophysiology, since not only acute moderate-to-severe symptomatic hyponatremia but also chronic minimal/mild symptomatic hyponatremia are associated with unfavorable outcomes [3–9]. Therefore, this review aimed to provide a detailed overview of hyponatremia management to support clinicians in optimizing treatment strategies in clinical practice.

## Treatment of hyponatremia

### Definition of hyponatremia

Hyponatremia is classified into different categories according to the duration after disease onset (acute or chronic), serum sodium concentration ([Na^+^]) (mild, moderate, or severe), and the severity of neurological symptoms (no or minimal/mild, moderate, or severe). Clinicians must understand these three classifications not only for diagnosis but also for treatment (Tables 1 and 2) [10, 11].

**Table 1:** Definitions of hyponatremia (modified from previous studies [10, 11])

Definition by biochemical test value		Definition by duration from onset		Definition by severity of neurological symptoms	
Mild	130 ≤ , < 135 (mmol/L)	Acute	< 48 h	No or Minimal/Mild	Absence or Presence of minimal/mild symptoms
Moderate	125 ≤ , < 130 (mmol/L)	Chronic	≥ 48 h (or unknown)	Moderate	Presence of moderate symptoms
Severe	< 125 (mmol/L)			Severe	Presence of severe symptoms

**Table 2:** Neurological symptoms of hyponatremia by severity (modified from a previous study [11])

Severity	Neurological symptoms
No or Minimal/Mild	Difficulty concentrating, irritability, altered mood, depression, unexplained headache
Moderate	Altered mental status, disorientation, confusion, unexplained nausea, gait instability
Severe	Coma, obtundation, seizures, respiratory distress, vomiting

### Common principles of treatment of hyponatremia

Treatment of hyponatremia is usually more challenging than that of other electrolyte disorders due to its complexity and varying treatment goals and methods recommended by different guidelines [10, 12]. However, understanding the principles of treatment is crucial because of many commonalities across different guidelines and recommendations. The six principles of treatment are as follows:

1. Correct promptly with hypertonic saline (usually 3% saline) if acute and symptomatic (moderate-to-severe).
2. If chronic/asymptomatic or minimally/mildly symptomatic, correct at a moderate rate, regardless of the correction method.
3. Discontinue medications associated with impaired renal free water excretion or natriuresis.
4. Adhere to upper correction limit if in patients at high risk for developing osmotic demyelination syndrome (ODS), except in acute/symptomatic (moderate-to-severe) cases.
5. If overly rapidly corrected, re-lower to low serum [Na^+^] with 5% dextrose (D5W) and/or desmopressin in carefully selected patients.
6. Perform frequent blood tests and monitor urine output after treatment initiation.

**Fig. 1:**
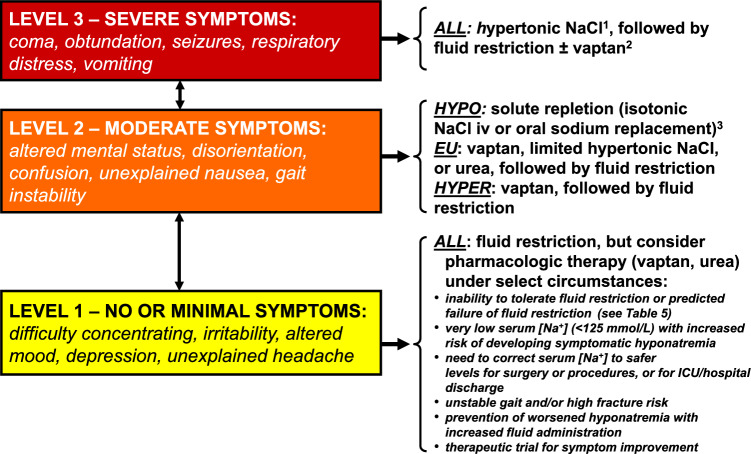
Hyponatremia treatment algorithm based on neurological symptoms (modified from a previous study [11]). The arrows between the symptom boxes indicate movement of patients between different symptom levels. ^1^Some authors recommend simultaneous treatment with desmopressin to limit speed of serum [Na^+^] correction. ^2^No active therapy should be started within 24 h of hypertonic saline to decrease the chance of overly rapid correction of serum [Na^+^] and risk of ODS. ^3^With isotonic saline infusion, serum [Na^+^] must be followed closely to prevent overly rapid correction and risk of ODS due to secondary aquaresis. Abbreviations: *ALL*, all types of hypotonic hyponatremia; *EU* euvolemic; *HYPER* hypervolemic, *HYPO* hypovolemic, *iv* intravenously, *NaCl* sodium chloride, *ODS* osmotic demyelination syndrome

If acute/symptomatic (moderate-to-severe) symptoms are suspected, treatment should be initiated with 3% saline (Fig. 1) [11]. Overly rapid correction can be prevented by closely monitoring the serum [Na^+^] and the urine output after treatment initiation, even if hyponatremia is not acute/symptomatic (moderate-to-severe). Additionally, even if an overly rapid correction occurs, there should be no concern regarding treating hyponatremia excessively, since re-lowering to low serum [Na^+^] can reduce the risk of developing ODS [13].

When treating patients with hyponatremia, it is practical to assess extracellular fluid volume and urine osmolality (U_Osm_) simultaneously, with understanding the mechanisms of urinary dilution and concentration in the context of treatment. Renal electrolyte-free water excretion is necessary to increase the serum [Na^+^]. Therefore, treatment should be directed toward eliminating any factors that contribute to impaired urinary dilution, and intentionally impairing the urinary concentrating mechanism is warranted (Table 3).

**Table 3:** Mechanisms of urinary dilution and concentration and their relationship to the treatment of hyponatremia

	Mechanisms	Treatment for hyponatremia
Urinary dilution	(1) Sufficient urinary flow to the ascending limb of the loop of Henle, the dilution site (2) Reabsorption of solutes, i.e., Na^+^ and Cl^−^, at the dilution site (3) No AVP activation of V2 receptors	Urinary dilution is impaired in hyponatremia and must be improved (1) Eliminate factors contributing to declined eGFR (2) Normalize ECF volume if hypovolemic (3) Discontinue natriuretics (4) Use V2 receptor antagonists
Urinary concentration	(1) Maintain hyperosmolality in the interstitium in the renal medulla (2) AVP is synthesized and secreted normally and binds to and acts on the V2 receptor in the renal collecting ducts, causing AQP2 to be inserted into the apical lumen of the renal collecting ducts	Intentionally impair urinary concentrating ability when treating hyponatremia (1) Administer urea and/or NaCl (2) Use V2 receptor antagonists

Abbreviations: *AQP* aquaporin, *AVP* arginine vasopressin, *ECF* extracellular fluid, *eGFR* estimated glomerular filtration rate, *NaCl* sodium chloride

### Treatment goals and upper correction limits of hyponatremia: not identical between the U.S. Expert Panel Recommendations and the European Clinical Practice Guideline

#### Acute/symptomatic (moderate-to-severe) hyponatremia

The treatment principle of acute/symptomatic hyponatremia is to promptly elevate serum [Na^+^] to decrease cerebral edema and improve neurological symptoms. Here, we compare the U.S. Expert Panel Recommendations and the European Clinical Practice Guideline for treatment goals (Table 4) [10, 12]. The treatment goals for acute/symptomatic (moderate-to-severe) hyponatremia were not significantly different between the two groups. A 4–6 mmol/L increase in serum [Na^+^] is considered sufficient to decrease intracranial pressure and prevent severe outcomes due to cerebral edema and brain herniation [14, 15]. Therefore, treatment generally targets an increase of approximately 5 mmol/L to resolve symptoms. As a significant difference, the U.S. Expert Panel Recommendations do not set an upper limit of Δserum [Na^+^], whereas the European Clinical Practice Guideline set an upper limit with Δserum [Na^+^] < + 10 mmol/L/d. Therefore, since treatment aims to improve cerebral edema and resolve symptoms, increasing serum [Na^+^] more than needed is unnecessary once symptoms are resolved.

**Table 4:** Correction rates of serum [Na^+^] (modified from previous studies [10, 12])

	U.S. Recommendations	European Guideline
Acute/symptomatic (moderate-to-severe)	Goal: + 4 to 6 mmol/L/d Upper correction limit: none	Goal: + 5 mmol/L/d Upper correction limit: + 10 mmol/L/d
Chronic/asymptomatic or symptomatic (minimal/mild)	Goals: + 4 to 8 mmol/L/d, + 4 to 6 mmol/L/d (ODS high risk) Upper correction limits: + 10 to 12 mmol/L/d, + 8 mmol/L/d (ODS high risk)	Goal: none Upper correction limit: + 10 mmol/L/d

Abbreviations: *ODS* osmotic demyelination syndrome

However, if no non-osmotic arginine vasopressin (AVP) secretion occurs (i.e., appropriate osmotic suppression), as observed in acute water intoxication, serum [Na^+^] may rapidly improve to normal concentrations with a large amount of diluted urine excretion after the treatment initiation. Contextually, the U.S. Expert Panel Recommendations state that “true” acute hyponatremia does not require an upper limit of Δserum [Na^+^] [12], although determining “true” symptomatic hyponatremia is sometimes difficult. Therefore, treating symptomatic hyponatremia is reasonable with 3% saline and an upper limit of Δserum [Na^+^]. Conversely, the European Clinical Practice Guideline set the above-mentioned upper correction limits [10]. However, the European Clinical Practice Guidelines face a challenge in determining the approach to evaluate and treat cases where severe symptoms associated with “true” symptomatic hyponatremia appear to persist even after the correction reaches its upper limit.

#### Chronic/asymptomatic or symptomatic (minimal/mild-to-moderate) hyponatremia

Preventing ODS development is essential in treating chronic/asymptomatic or symptomatic (minimal/mild to moderate) hyponatremia compared to acute/symptomatic hyponatremia; therefore, serum [Na^+^] should not be raised rapidly. Chronic/asymptomatic or minimally/mildly to moderately symptomatic hyponatremia differs from acute/symptomatic hyponatremia in that it carries a lower risk of short-term severe complications. Therefore, the U.S. Expert Panel Recommendations and the European Clinical Practice Guideline set upper correction limits. However, treatment targets are placed in the U.S. Expert Panel Recommendations but not in the European Clinical Practice Guideline (Table 4) [10, 12]. Although aggressive correction for chronic/asymptomatic or minimally/mildly to moderately symptomatic hyponatremia has not been shown to improve prognosis, it should be corrected steadily because the long-term prognosis is poor [16]. In chronic/asymptomatic or minimally/mildly to moderately symptomatic hyponatremia, fluid restriction, increased solute intake, and discontinuation of offending medications may sometimes be sufficient to improve the condition. Therefore, these strategies should be instituted in the early stages of treatment, although fluid restriction alone rarely improves hyponatremia sufficiently [17]. In many cases, low serum [Na^+^] are acceptable even if not elevated early in treatment while awaiting responses to more conservative strategies, since ODS development must be prevented [18]. In such cases, more aggressive treatments (e.g., 3% saline, vaptans, urea) can be withheld after confirming that the serum [Na^+^] is not corrected adequately with conservative treatment alone.

### Treatment options for hyponatremia

#### Hypertonic saline (3% saline or higher)

**Fig. 2:**
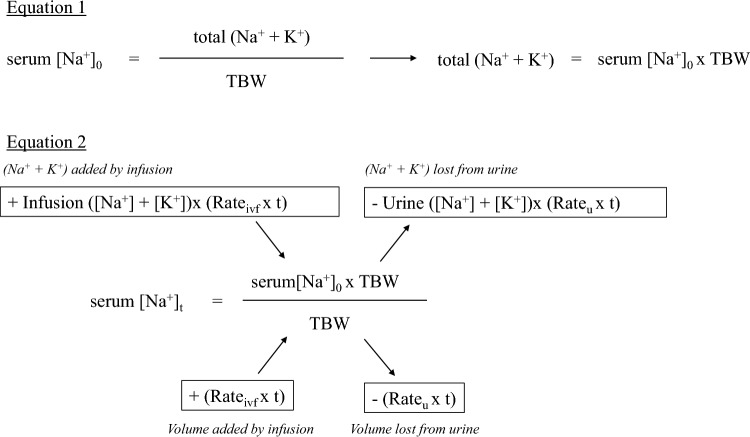
Prediction equation for correction of hyponatremia using the Edelman equation (modified from a previous study [22]). In Eq. 1, the total (Na^+^ + K^+^) in the body before treatment is calculated using the Edelman equation. In Eq. 2, the serum [Na^+^] after treatment is predicted if the volume of infusion and its composition, as well as the volume of urine and its composition, are known. For example, if a patient with serum [Na^+^]_0_ of 120 mmol/L, TBW of 50 L, and urine ([Na^+^] + [K^+^]) of 300 mmol/L is urinating at 50 mL/h, and isotonic saline [([Na^+^] + [K^+^]) = 154 mmol/L] is administered at 100 mL/h, we can calculate the predicted serum [Na^+^] after 12 h (assuming that the amount of urine and its composition remain unchanged). Substituting each into Eq. 2 yields 118.7 mmol/L. If the infusion fluid is hypertonic saline [for example, ([Na^+^] + [K^+^]) = 308 mmol/L], which is twice the concentration of normal saline, and is calculated under the same conditions, the predicted serum [Na^+^] is 122.3 mmol/L. Therefore, it is essential to administer infusions that are more hypertonic than urine ([Na^+^] + [K^+^]) to effectively increase the serum [Na^+^]. Abbreviations: serum [Na^+^]_0_, serum [Na^+^] before treatment; serum [Na^+^]_t_, predicted serum [Na^+^] after t h; Rate_ivf_, fluid volume per hour (L/h); Rate_u_, urine volume per hour (L/h); *TBW* total body water (L)

The [Na^+^] of 3% saline is 513 mmol/L, and the urine tonicity of humans cannot exceed the tonicity of 3% saline; therefore, administering 3% saline certainly will elevate the serum [Na^+^]. Recent reports have generally recommended bolus administration of 3% saline [19, 20], although whether bolus or continuous administration is better for increasing serum [Na^+^] remains uncertain. A prospective study in Ireland comparing patients administered boluses (100 mL) versus continuous (20 mL/h) 3% saline for hyponatremia due to SIAD showed a significantly early elevation in Δserum [Na^+^] and improvement in the Glasgow Coma Scale score 6 h after administration in the bolus group [19]. In the SALSA trial, a randomized control trial (RCT) conducted in South Korea that enrolled patients with moderately severe to severe hyponatremia and serum [Na^+^] ≤ 125 mmol/L, no significant differences were noted in efficacy and safety between the bolus [2 mL/kg body weight (BW)] and continuous (0.5–1 mL/kg BW/h) administration. The bolus group had a lower proportion of re-lowering for overly rapid correction than the continuous group (41.4% vs. 57.1%; absolute risk difference, – 15.8% [95% CI, – 30.3% to – 1.3%]; *P* = 0.04) [20]. For the bolus dose, the U.S. Expert Panel Recommendations and the European Clinical Practice Guideline set the volumes to 100 and 150 mL, respectively [10, 12]. However, a retrospective observational study involving patients with symptomatic hyponatremia who received a bolus dose (100 or 150 mL) of 3% saline reported that low BW (≤ 60 kg) was associated with an overly rapid correction [21]. Considering that the bolus dose was adjusted by BW in the SALSA study to prevent an overly rapid correction, a reduced dose of 1–2 mL/kg BW can be considered for ethnic groups with smaller body sizes, such as Asians, compared to that for Europeans and North Americans. Regardless of the method selected, monitoring serum [Na^+^], urine output, and U_Osm_ or urine specific gravity frequently (every 2–4 h) is more crucial. A recent report showed that the Edelman equation is useful for predicting the increase in serum [Na^+^] (Fig. 2) [22].

#### Fluid restriction and solute loading

As evident from the Edelman equation [23, 24], the serum [Na^+^] can be increased by decreasing the denominator, total body water, and increasing the numerators, Na^+^ and potassium (K^+^). Solute loading increases renal free water excretion because urine volume is defined as daily urinary solute excretion/U_Osm_ [25]. Therefore, fluid restriction and solute loading are theoretically appropriate, and fluid restriction is usually the first-choice treatment in guidelines for treatment of chronic hyponatremia [10, 12].

Table 5 presents the general recommendations for fluid restriction and the factors contributing to treatment resistance [12]. Although fluid restriction is associated with fewer complications and is generally well tolerated [12], it is usually relatively ineffective in many cases and may require a combination with other treatments [17]. In an RCT comparing fluid restriction (≤ 1 L/d) without specific treatment for SIAD due to chronic moderate hyponatremia, median plasma [Na^+^] increased from baseline by 4 mmol/L [interquartile range (IQR) 2–6] with fluid restriction, compared with 1 mmol/L (IQR 0–1) with no specific treatment (P = 0.04). However, there was not a great difference with respect to absolute elevation in serum [Na^+^] between the groups [17].

**Table 5:** General recommendations for fluid restriction and factors contributing to treatment resistance (modified from a previous study [12])

*General recommendations*
Restrict all kinds of fluid intake that are consumed through drinking, not just water Aim for a fluid restriction that is 500 mL/d below the 24-h urine volume Do not restrict sodium or protein intake unless indicated
*Predictors of failure of fluid restriction*
High U_Osm_ (> 500 mOsm/kg H_2_O) The urine ([Na^+^] + [K^+^]) exceeds the serum [Na^+^] 24-h urine volume < 1500 mL/d Increase in serum [Na^+^] < 2 mmol/L/d in 24–48 h on a fluid restriction of 1 L/d

Abbreviations: *U*_*Osm*_, urine osmolality

As previously mentioned, “effective osmolality in the apical lumen of the renal collecting duct less than that in the renal medullary interstitium” is required for free water reabsorption. Conversely, renal free water excretion increases when the effective osmolality in the apical lumen of the renal collecting duct is greater than that in the renal medullary interstitium; this is termed solute diuresis (osmotic diuresis). In solute diuresis, solute loading should increase the amount of solute reaching the renal collecting ducts and the effective osmolality within the apical lumen of the renal collecting ducts. Solute loading options include sodium and urea ingestion. Oral urea is a crystalline powder administered at a dose of 15–60 g/d. However, due to its bitter taste, it is advisable to dissolve it in sweet-tasting liquids [10]. A recent small study for outpatients with chronic SIAD (the TREASURE study) also suggested that a 7-day dietary protein supplementation of 90 g/d may be efficacious in increasing plasma [Na^+^] through protein-induced ureagenesis leading to osmotic diuresis followed by electrolyte-free water clearance [26]. When comparing the solute load for ingesting 600 mg of salt (1 tablet) with 15 g of urea (1 sachet), equal to 21 mOsm of salt and 250 mOsm of urea, leading to a significant difference in free water excretion of 42 and 500 mL, respectively, for urine tonicity of 500 mOsm/kg H_2_O. Thus, urea is superior to sodium in terms of efficacy [9]. However, no RCTs on the efficacy of urea are currently available, and only few observational studies have been conducted. A relatively large observational study of 69 patients with 78 occasions of any duration of hyponatremia (median nadir serum [Na^+^] 122 mmol/L) treated with urea showed that 64.1% of patients achieved serum [Na^+^] of ≥ 130 mmol/L in 72 h [27]. Urea dosages vary according to the guidelines; however, starting at 30 g/d is common. In a recent observational study involving inpatients with hyponatremia (plasma [Na^+^] < 135 mmol/L), 161 treatment episodes of urea were administered to 140 patients, with 93% diagnosed with SIAD. Patients received a median dose of 30 g/d of urea for 4 days, either in combination with fluid restriction or alone. The resulting serum [Na^+^] increased by 7 mmol/L over the 4-day period, with normalization of serum [Na^+^] occurring in 47% of treatment episodes, while overly rapid correction was noted in only 3% (5 cases). In the five cases of overly rapid correction, oral urea and fluid restriction were discontinued in four and three patients, respectively. Additionally, three of these five patients received plasma [Na^+^] re-lowering therapy (two with hypotonic fluids only and one with hypotonic fluids plus desmopressin) [28]. The increase in urine volume due to oral urea is determined by dividing the increase in urea excretion by urine osmolality. If AVP secretion is suppressed and urine osmolality falls, the resultant large volume of dilute urine output can lead to a greater rise in serum [Na^+^] than anticipated [29]. Notably, older age, lower baseline plasma [Na^+^], and higher cumulative urea dose were independently associated with a greater increase in plasma [Na^+^]. Furthermore, a sub-analysis of the first 48 h of treatment with oral urea indicated that concomitant fluid restriction was independently associated with a rise in plasma [Na^+^]. Therefore, clinicians should be aware of the potential for overly rapid correction in applicable patients [28]. The effect of oral urea in correcting hyponatremia has been demonstrated as described above, however, it is important to note that urea is not currently approved as a drug in Japan or the U.S. Although sodium chloride (NaCl) intake may theoretically improve hyponatremia, no studies have demonstrated the efficacy of NaCl intake alone, with only the EFFUSE-FLUID Trial, an RCT comparing fluid restriction with fluid restriction plus furosemide or fluid restriction plus furosemide plus salt intake for chronic hyponatremia due to SIAD [30]. However, this study did not show a significant benefit of any treatment group compared with that of the fluid restriction-only group on day 4 (Δserum [Na^+^]: fluid restriction 4.9 ± 4.7, fluid restriction plus furosemide 4.3 ± 4.4, fluid restriction plus furosemide plus salt intake 6.3 ± 8.7 mmol/L; *P* = 0.7). Additionally, the furosemide group experienced an increased frequency of hypokalemia (serum [K^+^] ≤ 3.0 mmol/L; *P* = 0.01) [30]. Furthermore, a trend toward an increased frequency of acute kidney injury was observed in the furosemide group and among patients fluid-restricted to 800 mL/d without statistical difference.

Sodium-glucose cotransporter (SGLT) 2 inhibitors have been approved as oral medications for type 2 diabetes, and more recently for treatment of chronic heart failure and advanced chronic kidney disease. They inhibit SGLT2 in the renal proximal tubules and increase glucose excretion into the urine, resulting in a solute (osmotic) diuresis and a secondary increase in free water excretion. In two RCTs comparing empagliflozin, an SGLT2 inhibitor, with placebo, the SGLT2 inhibitor significantly increased serum [Na^+^] in hyponatremia due to SIAD compared with placebo, but with an effect size of only 4.1 mmol/L [31, 32]. However, SGLT2 inhibitors should be avoided in patients with poor general conditions because of the risk of diabetic ketoacidosis, particularly in hospitalized patients with type 2 diabetes [33]. In addition, SGLT2 inhibitors are currently not approved as a medication for treatment of hyponatremia.

#### Aquaretic agents

Vasopressin V2 receptor (V2R) antagonists (called “vaptans”) cause a solute-free water diuresis, which is more appropriately termed “aquaresis”, by inhibiting the AVP activity in the renal collecting duct. Tolvaptan, an oral vasopressin V2R antagonist, is the only evidence-based medication for treating hyponatremia with efficacy demonstrated through RCTs. Tolvaptan has been shown to elevate serum [Na^+^] in hyponatremia observed in patients with heart failure, cirrhosis, SIAD, and malignancy, compared to placebo [34–36]. However, many post-marketing observational studies have reported a risk of overly rapid correction by tolvaptan use [37, 38]. The risks of overly rapid correction resulting from tolvaptan include low initial serum [Na^+^], comorbid malignancy, low body mass index, low blood urea nitrogen, and receiving other treatments for hyponatremia [9]. Tolvaptan-induced overly rapid correction of hyponatremia reportedly occurs even at a low dose of 3.75 mg/d; therefore, strict monitoring of serum [Na^+^] is required during treatment. However, it should be noted that most studies reporting overly rapid correction of serum [Na^+^] with tolvaptan used a cutoff of > 8 mmol/L/24 h as the criterion for overly rapid correction, and none reported the occurrence of ODS in patients corrected > 8 mmol/L/24 h. Table 6 summarizes the precautions required when using tolvaptan to correct hyponatremia.

**Table 6:** Cautions in the use of tolvaptan (modified from a previous study [9])

Exclude other causes of hyponatremia, particularly hypovolemia
Avoid using in patients with end-stage kidney disease or substantial severe liver dysfunction.^*^
Do not use in combination with other effective treatments for hyponatremia
Ensure free access to water once tolvaptan is administered (no fluid restriction), and ensure that patients are physically and cognitively able to drink unaided (and/or ability to provide IV fluid if required for re-lowering against overly rapid correction)
A low initial dose of tolvaptan 7.5 mg is preferred
Give tolvaptan early in the morning if possible; therefore, most of the aquaresis occurs in the afternoon/evening rather than overnight (to ease monitoring and minimize patient sleep disruption)
Monitor fluid intake and urine output carefully (with or without indwelling urinary catheter)
Recheck serum [Na^+^] 6 and 12 h after tolvaptan dosing
If serum [Na^+^] increases by > 6 mmol/L within 6 h, or 8 mmol/L within 12 h, initiate preemptive treatment with D5W matched to the rate of urine output to ameliorate further serum [Na^+^] elevation, with ongoing frequent monitoring of serum [Na^+^]. If serum [Na^+^] subsequently reduces to within these targets, D5W should be paused

*Tolvaptan 7.5 mg once daily is indicated in Japan for the treatment of ascites caused by liver cirrhosis

Abbreviations: *D5W* dextrose 5%, *IV* intravenous

### Prevention of ODS and management of overly rapid correction of hyponatremia

ODS is a severe complication that occurs when an overly rapid correction is caused in patients with chronic hyponatremia. The symptoms, including dysarthria, dysphagia, tetraplegia, disorientation, and coma, are usually irreversible, but depend on the severity of the initial demyelination [39]. ODS usually develops 2–6 days after overly rapid correction and should be suspected when symptoms reappear after initial improvement of symptom due to hyponatremia. Corticosteroids, immunoglobulins, and plasma exchange therapy have occasionally been used to treat ODS. However, no clear evidence currently exists to support such treatments; therefore, the most important objective is to prevent ODS onset [9]. Specifically, overly rapid correction in patients at high risk of developing ODS, e.g., serum [Na^+^] ≤ 105 mmol/L, hypokalemia, malnutrition, alcoholism, severe liver disease, should be carefully considered [12]. As indicated by K^+^ in the Edelman equation, supplemental K^+^ is also a factor that can elevate the serum [Na^+^] [40]. A case report described a patient with hypokalemia, without hyponatremia but with multiple ODS risk factors, who developed ODS during treatment to correct serum [K^+^] [41]. Although this is a rare case, overly rapid correction is common in patients with hyponatremia and concomitant hypokalemia, particularly due to thiazide use. Using the Edelman equation to treat hyponatremia can reduce the frequency of overly rapid corrections and ensure an appropriate correction rate [42]. Therefore, the Edelman equation should be used to predict response to therapy, particularly in patients with concomitant hypokalemia, but with the limitation that all equations fail to include the effect of a secondary aquaresis to rapidly increase the serum [Na^+^].

An observational study of 1490 patients with admission serum [Na^+^] < 120 mmol/L showed that overly rapid correction (Δserum [Na^+^] > + 8 mmol/L in 24 h) occurred in as many as 41% of the patients, but ODS occurred in only 0.5% of the hyponatremic patients [43]. The study also showed the following risk factors for overly rapid correction of hyponatremia: young age (63 vs. 68 years), female sex, schizophrenia, low Charlson Comorbidity Index, low serum [Na^+^] at the initial visit, and urine [Na^+^] < 30 mmol/L [43]. Among these risk factors, which are often experienced in clinical practice, low urine [Na^+^] is compellingly associated with overly rapid correction of hyponatremia. This association is attributed to the overly rapid correction that occurs when large volumes of diluted urine are excreted as aquaresis [10, 12]. The European Clinical Practice Guideline also states that a risk of overly rapid correction exists when urine output suddenly increases to > 100 mL/h, and that serum [Na^+^] and urine output should be measured every 2 h until the condition stabilizes [10]. A more recent retrospective analysis of 22,858 hospitalizations over 10 years with [Na^+^] < 130 mmol/L similarly showed a high rate of correction > 8 mmol/L (17.7%) but a low rate of ODS (0.05%) [44]. This has led to a reconsideration of the recommended rates of hyponatremia correction, but until more definitive studies are conducted, current expert opinion still recommends correcting cautiously in patients with a serum [Na^+^] < 120 mmol/L: correction of serum [Na^+^] by ≤ 10 mmol/L within 24 h or by ≤ 18 mmol/L within 48 h in most patients, but correction by ≤ 8 mmol/L within 24 h in patients with additional risk factors for ODS [45].

The three treatment strategies for overly rapid correction of hyponatremia are proactive, reactive, and rescue [46]. Proactive is the intervention before the serum [Na^+^] elevates, and reactive is when the serum [Na^+^] elevates, urine volume increases, or U_Osm_ falls. A proactive strategy involves using desmopressin to render the patient antidiuretic while infusing 3% saline at a rate predicted to increase serum [Na^+^] safely using the Adrogue-Madias formula [47]. This method, sometimes called the “Sterns protocol,” avoids overly rapid correction due to secondary aquaresis [48]. Reactive strategies involve stopping the correction once the desired correction limit is achieved. This can usually be accomplished by replacing ongoing urine output with equivalent volumes of D5W on an hourly basis. However, if the aquaresis is pronounced, desmopressin (2 µg intravenously or subcutaneously every 6–8 h) can be used to decrease the urine output. The U.S. Expert Panel Recommendations describe the rescue strategy as a method for re-lowering the overly corrected serum [Na^+^] to the upper correction limit. They suggest considering re-lowering in patients with hyponatremia with low-to-moderate ODS risk and recommend more aggressive re-lowering in patients with hyponatremia with high ODS risk [12]. The standard methods of re-lowering are intravenous desmopressin and continuous D5W administration [12]. Desmopressin, a V2R agonist, not only inhibits aquaresis but also promotes hemostasis by stimulating the Weibel–Palade bodies of endothelial cells to release von Willebrand factor and increase factor VIII levels [49, 50]. Thus, desmopressin is used not only for treating AVP deficiency (formerly central diabetes insipidus) but also for managing type 1 von Willebrand disease to prevent bleeding during minor invasive procedures [50]. Desmopressin used for overly rapid correction of hyponatremia treatment is usually administered at 2 µg every 6–8 h, although the duration varies among individuals, and the administration interval should be adjusted for each patient. Regarding the amount of D5W, the European Clinical Practice Guideline and U.S. Expert Recommendations recommend 10 and 3 mL/kg BW/h, respectively [10, 12]. Therefore, for more accurate re-lowering, D5W should be administered to lower serum [Na^+^] to the desired upper correction limit using the Adrogue–Madias formula [47] while stopping further urine output with desmopressin administration. Recent data suggest that these interventions should primarily be implemented in patients with severe hyponatremia (serum [Na^+^] ≤ 125 mmol/L) [46]. A recent single-center, open-label RCT to evaluate the safety and efficacy of proactive versus reactive desmopressin strategies in treating severe symptomatic hyponatremia with a serum [Na^+^] < 125 mmol/L revealed no significant difference in the incidence of the overly rapid correction (defined as a Δserum [Na^+^] > + 10 mmol/L in the first 24 h or > + 18 mmol/L in any 48 h in low-risk ODS patients, and > + 8 mmol/L in any 24 h in patients at high risk of ODS) between the proactive (16.7%) and reactive (28%) groups (*P* = 0.54) [51].

## Conclusions

Hyponatremia varies among patients and frequently exhibits a heterogeneous pathophysiology, posing difficulty in diagnosing the underlying cause and identifying the optimal treatment accordingly. However, since evidence in the management of hyponatremia is still insufficient, we hope that high-quality evidence based on RCTs comparing specific treatments will accumulate in the near future to enable individualized treatments for patients with hyponatremia. Until such time, we believe that this review will contribute to guiding best clinical practices in the management of hyponatremia.

## Data Availability

Not applicable.

## References

[1] Flear CT, Gill GV, Burn J. Hyponatraemia: mechanisms and management. Lancet. 1981;2:26–31. 6113402 10.1016/s0140-6736(81)90261-0

[2] DeVita MV, Gardenswartz MH, Konecky A, Zabetakis PM. Incidence and etiology of hyponatremia in an intensive care unit. Clin Nephrol. 1990;34:163–6. 2257702

[3] Portales-Castillo I, Sterns RH. Allostasis and the clinical manifestations of mild to moderate chronic hyponatremia: no good adaptation goes unpunished. Am J Kidney Dis. 2019;73:391–9. 30554800 10.1053/j.ajkd.2018.10.004

[4] Renneboog B, Musch W, Vandemergel X, Manto MU, Decaux G. Mild chronic hyponatremia is associated with falls, unsteadiness, and attention deficits. Am J Med. 2006;119(71):e1-8. 10.1016/j.amjmed.2005.09.026 16431193

[5] Verbalis JG, Barsony J, Sugimura Y, Tian Y, Adams DJ, Carter EA, Resnick HE. Hyponatremia-induced osteoporosis. J Bone Miner Res. 2010;25:554–63. 19751154 10.1359/jbmr.090827 PMC3153395

[6] Usala RL, Fernandez SJ, Mete M, Cowen L, Shara NM, Barsony J, Verbalis JG. Hyponatremia is associated with increased osteoporosis and bone fractures in a Large US health system population. J Clin Endocrinol Metab. 2015;100:3021–31. 26083821 10.1210/jc.2015-1261 PMC4524991

[7] Fujisawa H, Sugimura Y, Takagi H, Mizoguchi H, Takeuchi H, Izumida H, Nakashima K, Ochiai H, Takeuchi S, Kiyota A, Fukumoto K, Iwama S, Takagishi Y, Hayashi Y, Arima H, Komatsu Y, Murata Y, Oiso Y. Chronic hyponatremia causes neurologic and psychologic impairments. J Am Soc Nephrol. 2016;27:766–80. 26376860 10.1681/ASN.2014121196 PMC4769197

[8] Nowak KL, Yaffe K, Orwoll ES, Ix JH, You Z, Barrett-Connor E, Hoffman AR, Chonchol M. Serum sodium and cognition in older community-dwelling men. Clin J Am Soc Nephrol. 2018;13:366–74. 29439092 10.2215/CJN.07400717 PMC5967671

[9] Warren AM, Grossmann M, Christ-Crain M, Russell N. Syndrome of inappropriate antidiuresis: from pathophysiology to management. Endocr Rev. 2023;44:819–61. 36974717 10.1210/endrev/bnad010 PMC10502587

[10] Spasovski G, Vanholder R, Allolio B, Annane D, Ball S, Bichet D, Decaux G, Fenske W, Hoorn EJ, Ichai C, Joannidis M, Soupart A, Zietse R, Haller M, van der Veer S, Van Biesen W, Nagler E, Hyponatraemia Guideline Development Group. Clinical practice guideline on diagnosis and treatment of hyponatraemia. Eur J Endocrinol. 2014;170:1–47. 24569125 10.1530/EJE-13-1020

[11] Verbalis JG. Emergency management of acute and chronic hyponatremia. In: Matfin G, editor. Endocrine and metabolic emergencies. Washington: Endocrine Communications Press; 2014. p. 359.

[12] Verbalis JG, Goldsmith SR, Greenberg A, Korzelius C, Schrier RW, Sterns RH, Thompson CJ. Diagnosis, evaluation, and treatment of hyponatremia: expert panel recommendations. Am J Med. 2013;126(Supplement 1):S1-42. 24074529 10.1016/j.amjmed.2013.07.006

[13] Perianayagam A, Sterns RH, Silver SM, Grieff M, Mayo R, Hix J, Kouides R. DDAVP is effective in preventing and reversing inadvertent overcorrection of hyponatremia. Clin J Am Soc Nephrol. 2008;3:331–6. 18235152 10.2215/CJN.03190807 PMC2390955

[14] Sterns RH, Nigwekar SU, Hix JK. The treatment of hyponatremia. Semin Nephrol. 2009;29:282–99. 19523575 10.1016/j.semnephrol.2009.03.002

[15] Koenig MA, Bryan M, Lewin JL 3rd, Mirski MA, Geocadin RG, Stevens RD. Reversal of transtentorial herniation with hypertonic saline. Neurology. 2008;70:1023–9. 18272864 10.1212/01.wnl.0000304042.05557.60

[16] Hoorn EJ, Zietse R. Hyponatremia and mortality: moving beyond associations. Am J Kidney Dis. 2013;62:139–49. 23291150 10.1053/j.ajkd.2012.09.019

[17] Garrahy A, Galloway I, Hannon AM, Dineen R, O’Kelly P, Tormey WP, O’Reilly MW, Williams DJ, Sherlock M, Thompson CJ. Fluid restriction therapy for chronic SIAD; Results of a prospective randomized controlled trial. J Clin Endocrinol Metab. 2020;105:dgaa619. 32879954 10.1210/clinem/dgaa619

[18] Sterns RH. Adverse consequences of overly rapid correction of hyponatremia. Front Horm Res. 2019;52:130–42. 32097948 10.1159/000493243

[19] Garrahy A, Dineen R, Hannon AM, Cuesta M, Tormey W, Sherlock M, Thompson CJ. Continuous versus bolus infusion of hypertonic saline in the treatment of symptomatic hyponatremia caused by SIAD. J Clin Endocrinol Metab. 2019;104:3595–602. 30882872 10.1210/jc.2019-00044

[20] Baek SH, Jo YH, Ahn S, Medina-Liabres K, Oh YK, Lee JB, Kim S. Risk of overcorrection in rapid intermittent bolus vs slow continuous infusion therapies of hypertonic saline for patients with symptomatic hyponatremia: the SALSA randomized clinical trial. JAMA Intern Med. 2021;181:81–92. 33104189 10.1001/jamainternmed.2020.5519 PMC7589081

[21] Pelouto A, Refardt JC, Christ-Crain M, Zandbergen AAM, Hoorn EJ. Overcorrection and undercorrection with fixed dosing of bolus hypertonic saline for symptomatic hyponatremia. Eur J Endocrinol. 2023;188:322–30. 36881992 10.1093/ejendo/lvad028

[22] Chen S, Shey J, Chiaramonte R. Ratio profile: physiologic approach to estimating appropriate intravenous fluid rate to manage hyponatremia in the syndrome of inappropriate antidiuresis. Kidney360. 2022;3:2183–9. 36591355 10.34067/KID.0004882022 PMC9802565

[23] Edelman IS, Leibman J, O’Meara MP, Birkenfeld LW. Interrelations between serum sodium concentration, serum osmolarity and total exchangeable sodium, total exchangeable potassium and total body water. J Clin Invest. 1958;37:1236–56. 13575523 10.1172/JCI103712 PMC1062793

[24] Rose BD. New approach to disturbances in the plasma sodium concentration. Am J Med. 1986;81:1033–40. 3799631 10.1016/0002-9343(86)90401-8

[25] Berl T. Impact of solute intake on urine flow and water excretion. J Am Soc Nephrol. 2008;19:1076–8. 18337482 10.1681/ASN.2007091042

[26] Monnerat S, Atila C, Baur F, Santos de Jesus J, Refardt J, Dickenmann M, Christ-Crain M. Effect of protein supplementation on plasma sodium levels in the syndrome of inappropriate antidiuresis: a monocentric, open-label, proof-of-concept study-the TREASURE study. Eur J Endocrinol. 2023; 189:252–61. 10.1093/ejendo/lvad108 37540987

[27] Lockett J, Berkman KE, Dimeski G, Russell AW, Inder WJ. Urea treatment in fluid restriction-refractory hyponatraemia. Clin Endocrinol (Oxf). 2019;90:630–6. 30614552 10.1111/cen.13930

[28] Pelouto A, Monnerat S, Refardt J, Zandbergen AAM, Christ-Crain MC, Hoorn EJ. Clinical factors associated with hyponatremia correction during treatment with oral urea. Nephrol Dial Transplant. 2024;16:gfae164. 10.1093/ndt/gfae164 PMC11997808 39013606

[29] Sterns RH, Rondon-Berrios HB. Urea for hyponatremia: a role for desmopressin? Nephrol Dial Transplant. 2024;22:gfae69. 10.1093/ndt/gfae169 39039014

[30] Krisanapan P, Vongsanim S, Pin-On P, Ruengorn C, Noppakun K. Efficacy of furosemide, oral sodium chloride, and fluid restriction for treatment of syndrome of inappropriate antidiuresis (SIAD): an open-label randomized controlled study (The EFFUSE-FLUID Trial). Am J Kidney Dis. 2020;76:203–12. 32199708 10.1053/j.ajkd.2019.11.012

[31] Refardt J, Imber C, Sailer CO, Jeanloz N, Potasso L, Kutz A, Widmer A, Urwyler SA, Ebrahimi F, Vogt DR, Winzeler B, Christ-Crain M. A randomized trial of empagliflozin to increase plasma sodium levels in patients with the syndrome of inappropriate antidiuresis. J Am Soc Nephrol. 2020;31:615–24. 32019783 10.1681/ASN.2019090944 PMC7062212

[32] Refardt J, Imber C, Nobbenhuis R, Sailer CO, Haslbauer A, Monnerat S, Bathelt C, Vogt DR, Berres M, Winzeler B, Bridenbaugh SA, Christ-Crain M. Treatment effect of the SGLT2 inhibitor empagliflozin on chronic syndrome of inappropriate antidiuresis: results of a randomized, double-blind, placebo-controlled, crossover trial. J Am Soc Nephrol. 2023;34:322–32. 36396331 10.1681/ASN.2022050623 PMC10103093

[33] Hamblin PS, Wong R, Ekinci EI, Fourlanos S, Shah S, Jones AR, Hare MJL, Calder GL, Epa DS, George EM, Giri R, Kotowicz MA, Kyi M, Lafontaine N, MacIsaac RJ, Nolan BJ, O’Neal DN, Renouf D, Varadarajan S, Wong J, Xu S, Bach LA. SGLT2 inhibitors increase the risk of diabetic ketoacidosis developing in the community and during hospital admission. J Clin Endocrinol Metab. 2019;104:3077–87. 30835263 10.1210/jc.2019-00139

[34] Schrier RW, Gross P, Gheorghiade M, Berl T, Verbalis JG, Czerwiec FS, Orlandi C, SALT Investigators. Tolvaptan, a selective oral vasopressin V2-receptor antagonist, for hyponatremia. N Engl J Med. 2006;355:2099–112. 17105757 10.1056/NEJMoa065181

[35] Chen S, Zhao JJ, Tong NW, Guo XH, Qiu MC, Yang GY, Liu ZM, Ma JH, Zhang ZW, Gu F. Randomized, double blinded, placebo-controlled trial to evaluate the efficacy and safety of tolvaptan in Chinese patients with hyponatremia caused by SIADH. J Clin Pharmacol. 2014;54:1362–7. 24906029 10.1002/jcph.342

[36] Salahudeen AK, Ali N, George M, Lahoti A, Palla S. Tolvaptan in hospitalized cancer patients with hyponatremia: a double-blind, randomized, placebo-controlled clinical trial on efficacy and safety. Cancer. 2014;120:744–51. 24895288 10.1002/cncr.28468

[37] Arima H, Goto K, Motozawa T, Mouri M, Watanabe R, Hirano T, Ishikawa SE. Open-label, multicenter, dose-titration study to determine the efficacy and safety of tolvaptan in Japanese patients with hyponatremia secondary to syndrome of inappropriate secretion of antidiuretic hormone. Endocr J. 2021;68:17–29. 32863282 10.1507/endocrj.EJ20-0216

[38] Kleindienst A, Georgiev S, Schlaffer SM, Buchfelder M. Tolvaptan versus fluid restriction in the treatment of hyponatremia resulting from SIADH following pituitary surgery. J Endocr Soc. 2020;4:bvaa068. 32666012 10.1210/jendso/bvaa068 PMC7326480

[39] Singh TD, Fugate JE, Rabinstein AA. Central pontine and extrapontine myelinolysis: a systematic review. Eur J Neurol. 2014;21:1443–50. 25220878 10.1111/ene.12571

[40] Nguyen MK, Kurtz I. Role of potassium in hypokalemia-induced hyponatremia: lessons learned from the Edelman equation. Clin Exp Nephrol. 2004;8:98–102. 15235925 10.1007/s10157-004-0281-3

[41] Ormonde C, Cabral R, Serpa S. Osmotic demyelination syndrome in a patient with hypokalemia but no hyponatremia. Case Rep Nephrol. 2020;2020:3618763. 32274230 10.1155/2020/3618763 PMC7125463

[42] Nagase K, Watanabe T, Nomura A, Nagase FN, Iwasaki K, Nakamura Y, Ikai H, Yamamoto M, Murai Y, Yokoyama-Kokuryo W, Takizawa N, Shimizu H, Fujita Y. Predictive correction of serum sodium concentration with formulas derived from the Edelman equation in patients with severe hyponatremia. Sci Rep. 2023;13:1783. 36720979 10.1038/s41598-023-28380-y PMC9889706

[43] George JC, Zafar W, Bucaloiu ID, Chang AR. Risk factors and outcomes of rapid correction of severe hyponatremia. Clin J Am Soc Nephrol. 2018;13:984–92. 29871886 10.2215/CJN.13061117 PMC6032596

[44] MacMillan TE, Shin S, Topf J, Kwan JL, Weinerman A, Tang T, Raissi A, Koppula R, Razak F, Verma AA, Fralick M. Osmotic demyelination syndrome in patients hospitalized with hyponatremia. NEJM Evid. 2023; 2:EVIDoa2200215. 10.1056/EVIDoa2200215 38320046

[45] Sterns RH, Rondon-Berrios H, Adrogué HJ, Berl T, Burst V, Cohen DM, Christ-Crain M, Cuesta M, Decaux G, Emmett M, Garrahy A, Gankam-Kengne F, Hix JK, Hoorn EJ, Kamel KS, Madias NE, Peri A, Refardt J, Rosner MH, Sherlock M, Silver SM, Soupart A, Thompson CJ, Verbalis JG, PRONATREOUS Investigators. Treatment guidelines for hyponatremia: stay the course. Clin J Am Soc Nephrol. 2024;19:129–35. 37379081 10.2215/CJN.0000000000000244 PMC10843202

[46] MacMillan TE, Tang T, Cavalcanti RB. Desmopressin to prevent rapid sodium correction in severe hyponatremia: a systematic review. Am J Med. 2015;128(1362):e15-24. 10.1016/j.amjmed.2015.04.040 26031887

[47] Adrogué HJ, Madias NE. Hyponatremia. N Engl J Med. 2000;342:1581–9. 10824078 10.1056/NEJM200005253422107

[48] Sood L, Sterns RH, Hix JK, Silver SM, Chen L. Hypertonic saline and desmopressin: a simple strategy for safe correction of severe hyponatremia. Am J Kidney Dis. 2013;61:571–8. 23266328 10.1053/j.ajkd.2012.11.032

[49] Wang C, Lebedeva V, Yang J, Anih J, Park LJ, Paczkowski F, Roshanov PS. Desmopressin to reduce periprocedural bleeding and transfusion: a systematic review and meta-analysis. Perioper Med (Lond). 2024;13:5. 38263259 10.1186/s13741-023-00358-4 PMC10804695

[50] Seaman CD. Efficacy of adjusted weight-based dosing of desmopressin (1-deamino-8-d-arginine vasopressin) in type 1 von Willebrand disease. Blood Coagul Fibrinolysis. 2023;34:462–4. 37823430 10.1097/MBC.0000000000001251 PMC10836785

[51] Pakchotanon K, Kanjanasuphak N, Chuasuwan A, Gojaseni P, Chittinandana A. Safety and efficacy of proactive versus reactive administration of desmopressin in severe symptomatic hyponatremia: a randomized controlled trial. Sci Rep. 2024;14:7487. 38553491 10.1038/s41598-024-57657-z PMC10980789

